# The effect of ingestion of red dragon fruit extract on levels of malondialdehyde and superoxide dismutase after strenuous exercise in rats ( *Rattus norvegicus*)

**DOI:** 10.12688/f1000research.54254.2

**Published:** 2022-07-11

**Authors:** Gusbakti Rusip, Syafrudin Ilyas, I. Nyoman Ehrich Lister, Chrismis N. Ginting, Indra Mukti

**Affiliations:** 1 Department of Physiology, Faculty of Medicine, University Prima Indonesia, Medan, Sumatra Utara, 20118, Indonesia; 2 Department of Biology, Faculty of Mathematics and Natural Sciences, University Sumatera Utara, Medan, Sumatra Utara, 20132, Indonesia; 3 Universitas Prima Indonesia, Medan, Sumatera Utara, Indonesia; 4 Department of Surgery, Universitas Prima Indonesia, Medan, Sumatera Utara, Indonesia

**Keywords:** Red Dragon Fruit (RDF), Strenuous exercise, MDA, Improve function cell, Fatigue

## Abstract

**Background: **Prolonged activation of skeletal muscles causes a decrease in the production of fatigue. Exercise with strenuous intensity causes an increase in Reactive Oxygen Species (ROS). An increase in free radicals causes oxidative stress resulting in damage to cell function to mitochondrial dysfunction, and fatigue. This study aimed to determine the antioxidant potential of red dragon fruit (RDF) to delay fatigue due to oxidative stress, which improves cell function in mitochondria.

**Methods: **25 male rats *(* *Rattus norvegicus)* aged three months were divided into five groups: Group K1 was N.A. (No Activity) but drinking and eating; Group K2 performed strenuous exercise without RDF treatment; Groups 3, 4, and 5 (P1, P2 and P3, respectively) performed strenuous exercise and were treated with 75 mg kg ^-1^.bw, 150 mg kg ^-1^.bw, and 300 mg kg ^-1^.bw of RDF extract, respectively. The exercise for the rats involved intense swimming for 20 minutes every day, four days a week for 31 days.  Malondialdehyde (MDA) was measured with the ELISA and histopathology for muscle soleus and lung tissue.

**Results: **Strenuous exercise followed by RDF extract ingestion was compared for fatigue in terms of duration and time; before (24.55±1.38 minute) and after (95.31±7.82 minute) and led to a significant difference of 39% (p<0.01). The study also compared MDA before and after RDF extract ingestion in the K2 vs. the P1 group (p<0.05). At the same time, P2 differed more significantly (p<0.01). This indicated a spread of free radicals and featured histopathological damage of muscle cells. However, ingestion of RDF extract leads to improvement of soleus muscle cells; thus, repairs cell function, delaying fatigue.

**Conclusion: **This study confirmed that strenuous exercise, which causes an increase in ROS, intensifies free radicals with RDF extract ingestion and declines oxidative stress, repairing cell function and delaying fatigue.

## Introduction

Increased frequency, intensity, and duration of regular physical exercise improves performance and delays fatigue in daily work. ^1^ ^,^ ^2^ All living things, except those that are anaerobic, require oxygen to produce energy efficiently. Oxygen is an essential component of cellular metabolism. Exercise causes an increase in oxygen consumption by 10-12 times in the body, causing oxidative damage to the lipids of various tissues. ^3^ ^–^ ^7^ Moderate to high-intensity exercise can result in an increase in reactive oxygen species (ROS), free radicals in the body, which is characterized by an increase in malondialdehyde (MDA), and a decrease in superoxide dismutase (SOD) which is an endogenous antioxidant to suppress excess of free radicals. Several studies showed that reactive oxygen species (ROS) formed due to tissue hypoxia during muscle contraction have an adaptive physiological role during physical exercise. Oxidative stress is an imbalance between free radicals and antioxidants. Endogenous antioxidants cannot neutralize free radicals if they are formed excessively. ^8^ ^,^ ^9^ Oxidative stress causes damage to muscle cells and lungs, known as oxidative damage. It is the breakdown of biomolecules that make up cells due to reactions with free radicals. ^10^ Strenuous physical exercise will increase the growth of the free radicals found in muscle and liver tissue 2 to 3 times in experimental animals, which also will increase ROS. As a defense action, the body will be countered by the endogenous antioxidant system, ^11^ which is known as oxidative stress. This can be seen based on the ability of antioxidants in the tissue to neutralize ROS, ^12^ particularly the antioxidants produced by the body known as endogenous antioxidants that come from outside or exogenous antioxidants. These antioxidants come from food, such as fruit. Red dragon fruit (RDF) has been proven to protect the tissue from damage caused by ROS in the body. ^8^ ^,^ ^9^ ^,^ ^13^ This study aimed to examine effect of strenuous exercise on changes malondialdehyde and superoxide dismutase and by ingestion red dragon fruit extract improves MDA, SOD, muscle and lung tissue delay fatigue in rats ( *Rattus norvegicus*).

## Methods

### Animal experiment

Animal models, particularly rodents, are widely used in biological sciences, and the findings of animal research are traditionally projected to human response similar to physiological stimuli. ^14^ This article was reported in line with the ARRIVE guidelines. The study was a randomized post-test-only control group approved by the Animal Research Ethics Committee, Department of Biology - Faculty of Mathematics and Science, Universitas Sumatera Utara (approval number 0005/KEPH-FMIPA/2020).

In this study, we used 25 three-month-old male rats with an average weight of 200 g. The rats were obtained from the Animal House Unit of the Biology Laboratory, Universitas Sumatera Utara, Indonesia. All rats were maintained in groups in experimental animal cages in the laboratory. The cage (30 cm × 20 cm × 10 cm) was made of plastic and covered with fine wire mesh. The cage base was covered with rice husks with a thickness of 0.5–1 cm, which were replaced every day during the study. The room light was controlled to deliver a 12 h light/12 h dark cycle, the temperature was set to 25–27 °C, and the humidity of the room was adjusted to a normal range of 35–50%. The rats were fed standard rat pellets and given tap water *ad libitum.*

### Study design

We used an in vitro experimental method with a true experimental design and a randomised post-test for the control group. Simple random sampling was used to categorise the laboratory rats into five groups as follows: group K1 with no activity and no RDF; group K2 subjected to strenuous exercise without RDF (Red Dragon Fruit); and groups P1, P2, and P3 subjected to strenuous exercise and treated with 75, 150, and 300 mg kg ^−1^ body weight of RDF extract, respectively. In the fruit market, it is easy to find RFP fruit, acquired from farmers in Indonesia, was peeled, washed, cut into small pieces, and then dried in a drying cabinet. Next, the fruit was blended using a blender, and the extract was obtained by the maceration method with 96% ethanol, which was distilled by 10 times the weight of RDF. The RDF powder was stored in a container with 96% ethanol (ratio of 1:7, fruit powder: ethanol) and then soaked for 3 d. The RDF was macerated using a rotary evaporator at 45 °C until the extract thickened. The macerated RDF was extracted using 96% ethanol. The remaining extract was then evaporated in a water bath until a thick extract was obtained. Next, 100 mg RDF extract was weighed and crushed using a pestle and mortar. Subsequently, carboxymethylcellulose Na solution (0.5% w/v) was slowly added until a homogeneous extract was obtained, and the resulting volume was 10 mL. This final RFP extract was administered to the rats at appropriate dosages; specifically, rats weighing 200 g were fed 1.5, 3.0, or 6.0 mL of the RFP extract suspension, which corresponded to doses of 75, 150, or 300 mg kg ^−1^ body weight, respectively.

### Experimental procedures

The strenuous exercise given to all rats involved a morning swim between 08 – 09 AM for 20 minutes a day three times a week for four weeks. ^15^ The rats were treated with RDF extract every day for four weeks respectively at half an hour before the strenuous exercise. All rats completed the strenuous exercise test. At the end of the study, the results were obtained in the fourth week of exercise testing until the maximum exercise was swimming until almost drowning.

### Outcomes

One of the biomarkers of oxidative stress is a high level of malondialdehyde (MDA) and decreased SOD activity due to excessive lipid peroxidation processes in cells. One way to control excessive oxidative stress is by consuming antioxidants from food (exogenous antioxidants); one source of exogenous antioxidants is RDF, which consists of Group P1 treated with 75 mg kg ^−1^.bw; Group P2 with 150 mg kg ^−1^.bw; and Group P3 with 300 mg kg ^−1^.bw of RDF extract.

The consumption of RDF extract suppresses the increase in free radicals due to strenuous exercise. It increases SOD, an endogenous antioxidant, so oxidative stress does not occur, and repair mitochondrial cell function has fatigue delaying effect.

### Analysis of blood

All of the rats completed the strenuous exercise course. They experienced maximal physical activity, i.e., swimming, until they almost drowned. Blood for MDA and SOD was taken consecutively was assessed with enzyme-linked immune sorbent assay (ELISA) method and spectrophotometry with a wavelength of 450 nm. The assessment was done by using mouse malondialdehyde ELISA kit (Brand Bioassay TL, catalogue: EO625Mo). The SOD kit (Brand Bioassay TL, catalogue: EO168Ra) rat super oxidase dismutase ELISA kit was determined using the equation obtained from the standard curve. ^16^ ^–^ ^18^

### Histopathological study muscle tissue, and lung organs

Muscle soleus and lung tissue samples were collected by performing a biopsy to determine the degree of muscle damage based on haematoxylin and eosin (H&E) staining. The soleus muscle tissues of the rats were collected and fixed with 10% formalin for 24 h. The muscle and lung tissues were embedded in paraffin, sectioned to a 4 μm thickness, and stained via H&E staining. The stained sections were then examined under a light microscope (400× magnification) with 10 fields of view to determine the degree of damage concerning inflammatory cells and necrosis. The examination was conducted by a pathologist who was blinded to the applied treatment.

### Statistical analysis

Normality was assessed with Shapiro-Wilk test (p>0.05). Data Analysis was done by one-way analysis of variance (ANOVA) to indicate the effect of treatments for each group. The data were analysed with SPSS version 25 software and presented in tabulated and graphical forms as means and standard deviation. Significant differences were determined at p<0.05. The Post Hoc Bonferroni test was conducted after the significant results were obtained.

### Ethical approval

The animal subjects' research was performed according to the ethical standards by the Animal Research Ethics Committees/AREC, Faculty Mathematics and Natural Sciences Universitas Sumatera Utara, Indonesia (approval number 0005/KEPH-FMIPA/2021).

## Results

During consumption of the antioxidant RDF extract, all rats were accustomed to reducing stress-related disorders and seemed to be in good condition. No rats were poisoned, and there were no deaths in the experiment period.

A normality test indicated that the data are normally distributed ( Table 1).

**Table 1.** Normality for test MDA and SOD, with Shapiro-Wilk test, p>0.05.

Parameter	Group	Normality test	
		Statistic	p-value
Malondialdehyde (MDA)	K1	0.791	0.068
	K+	0.969	0.867
	P1	0.853	0.204
	P2	0.790	0.068
	P3	0.959	0.804
Superoxide dismutase (SOD)	K1	0.892	0.369
	K+	0.968	0.864
	P1	0.906	0.446
	P2	0.806	0.090
	P3	0.862	0.236

Strenuous exercise followed by RDF extract ingestion was compared for fatigue in terms of duration and time; before (24.55±1.38 minute) and after (95.31±7.82 minute) and led to a significant difference of 39% (p<0.01).

### The effects of strenuous exercise on MDA before and after RDF extract treatment

The results of One-Way ANOVA test for groups K2, P1, P2, and P3 showed significant differences ( Table 2). It is known that the measurement of MDA levels is a marker for assessing the increase in free radical production in rats treated with physical activity.

**Table 2.** Comparison of the MDA level results before and after treatment with RDF extract and the results of the One-Way ANOVA test.

Groups	MDA level (μg/dL)	p-value
K1	0.4191±0.2080 ^bc^	p<0.05
K2	0.5471±0.0399 ^c^	
P1	0.3120±0.1357 ^ab^	
P2	0.3159±0.0377 ^ab^	
P3	0.2531±0.0284 ^a^	

Notation. bc, c: p>0.05.

Notation. ab, ab: p>0.05.

Notation. c vs ab, ab, a: p<0.05.

**Figure 1.**
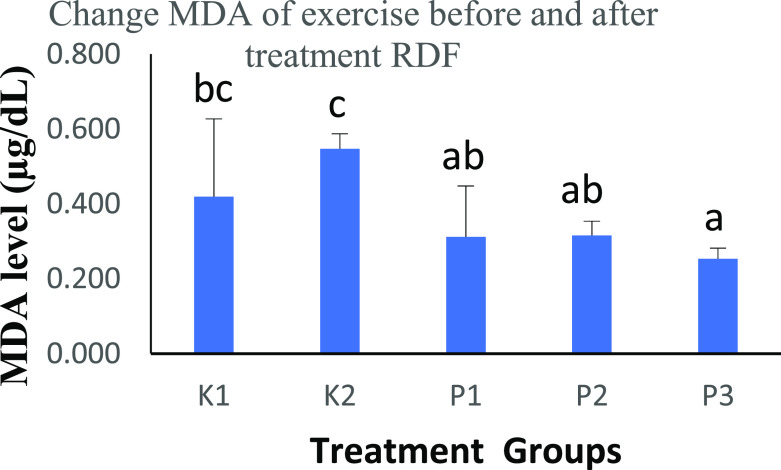
Graph changes of MDA levels (μg/dL) before and after treatment RDF extract; Mean SD. Note: The different notation letters on the bar graph are significantly different (p<0.05). Legend of figure: a bc = notation to see the difference is real or not, if a means the difference is not real. If a b = significant difference (p<0.05).

MDA expression ( Table 2 and Figure 1) was decreased after treatment with RDF extract (0.4191 vs 0.5471 vs 0.3120 vs 0.3159 vs 0.2531 μg/dL). The P3 group had the lowest score compared to the other groups. This study showed a significant reduction between groups.

The study results compared the MDA of rats after ingestion of RDF extract, which was tested with the Post Hoc test - Bonferroni. In the K2 vs. P1 group, there was a significant difference of p<0.05, the K2 vs. P2 group had a significant difference of p<0.05, and the K2 vs. P3 group had an increased significant difference of p<0.01.

### The effects of strenuous exercise on SOD before and after RDF extract treatment

**Figure 2.**
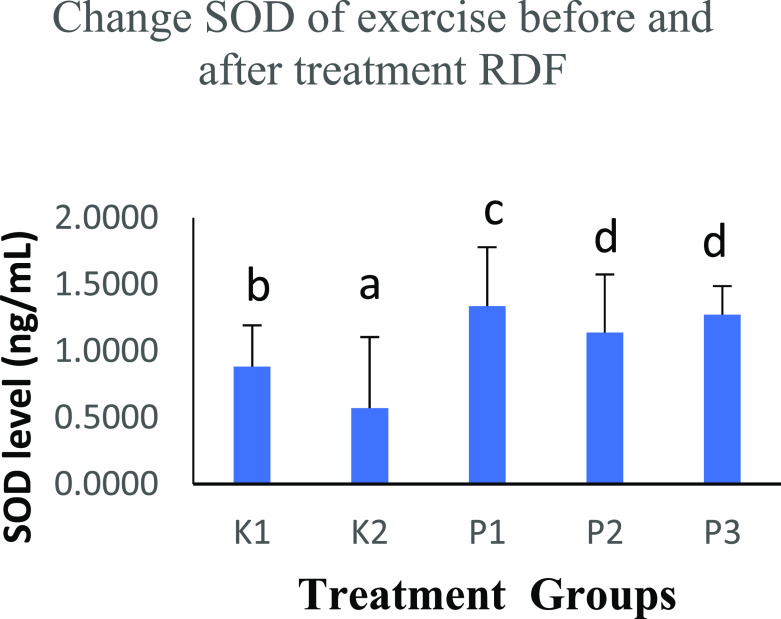
Graph changes of SOD levels (ng/mL) before and after treatment RDF extract. Note: The different notation letters on the bar graph are significantly different (p<0.05).

The free radicals in the body are balanced with endogenous defense mechanisms, and the body will produce antioxidants with an anti-free radical effect. In this study, the K2 group performed physical activity and SOD levels were 0.4632±0.2449 ng/mL. There was an increase in SOD levels in the K1 group (0.8647±0.1744 ng/mL) that did not perform physical activity. The increase in SOD continued with RDF extract treatment in groups K1 (1.3499±0.1359 ng/mL), P2 (1.9370±0.0236 ng/mL) and P3 (1.9521±0.0239 ng/mL). The three groups were given RDF treatment and showed significant differences (p<0.05), analysed with the One-Way ANOVA test ( Table 3 and Figure 2).

**Table 3.** Comparison of the results of the SOD level before and after treatment RDF extract.

Groups	SOD level (ng/mL)	p-value
K1	0.8647±0.1744 ^b^	p<0.05
K2	0.4632±0.2449 ^a^	
P1	1.3499±0.1359 ^c^	
P2	1.9370±0.0236 ^d^	
P3	1.9521±0.0239 ^d^	

Notation. a, b, c, d: p<0.05.

Notation. d, d: p>0.05.

### Histopathologic changes in muscle and lung tissue

**Figure 3.**
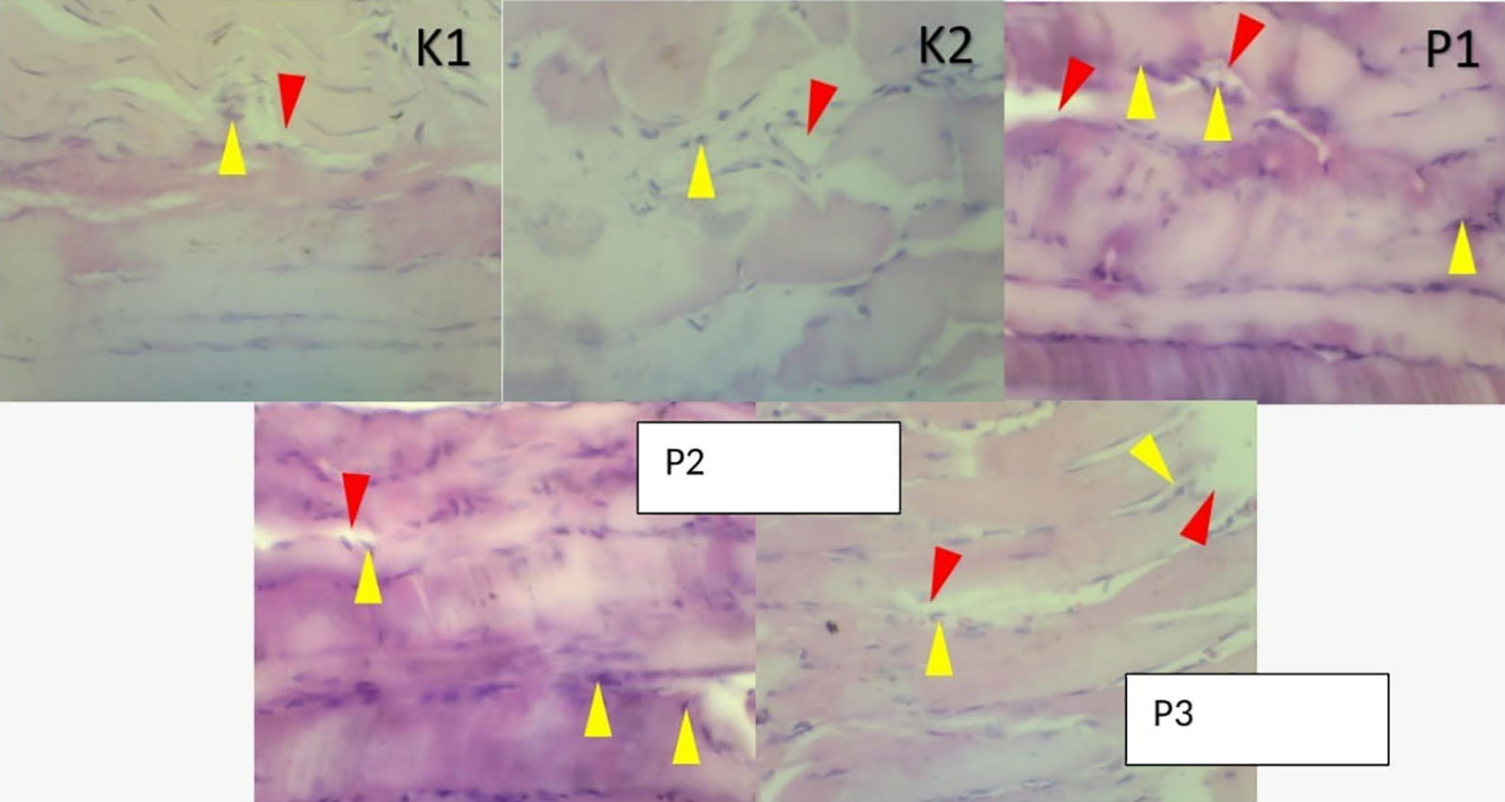
A picture of changes in muscle soleus cells before and after treatment RDF extract (antioxidant exogen). Arrow yellow: inflammatory cells, Arrow red: necrosis.

**Figure 4.**
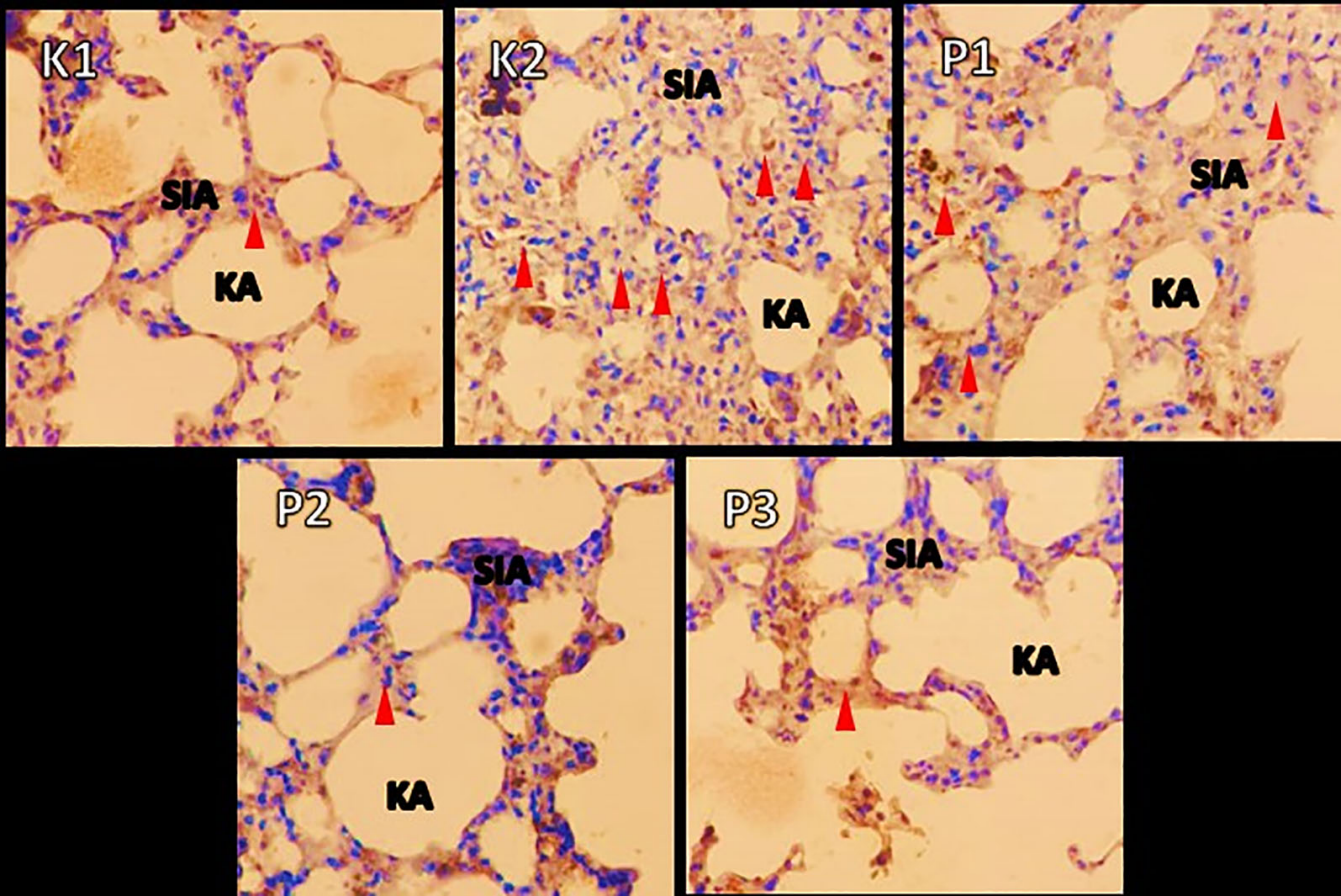
A picture of changes in the lung organs in the rat before and after treatment RDF extract (antioxidant exogen). Note: Red arrow = inflammatory cells; SIA = interalveolar septum, KA = alveolar sac.

The histopathological examination were observed under a microscope. It was seen that in group K1 changes in muscle and lung tissue did not occur. Ingroup K2 the changes were very significant, and many inflammatory cells and necrosis were observed in both the muscles and lungs. In contrast to P1, the P2 and P3 groups showed a decrease in inflammatory cells. In addition, in these two groups compared to P1, the lungs in the intra-alveolar and the alveolar sacs were dilated, and tissue repair was shown by the hyalinization process. Results showed changes in free radicals that could damage tissue in the positive control group K2. In contrast, the histopathological features of the P1, P2, P3 groups showed lung tissue and muscle cell repair, after being given RDF ( Figures 3 and 4).

## Discussion

Free radicals in the skeletal muscles cause muscle fatigue. The free radicals significantly reduce muscle strength, contributing to muscle fatigue during prolonged training. ^7^ ^,^ ^19^ ^,^ ^20^ The role of oxidants in muscle fatigue has been investigated in various animal models in vitro and situ during exercise. Oxidants are detectable in muscle at low levels during rest and at higher levels during contractions. RNS depress force production but do not appear to cause fatigue of healthy muscle. In contrast, muscle-derived ROS contribute to fatigue because loss of function can be delayed by ROS- specific antioxidants. ^21^ ^–^ ^23^ A study showed that exogenous antioxidants derived from food to capture ROS slowed down muscle fatigue, and enzymatic and nonenzymatic antioxidants delayed muscle fatigue during contraction. In the study, the subject characteristics have been standardized in accordance with WHO, adjusted to the provisions of the criteria ^24^ ^–^ ^26^ in the Research Guideline for Evaluating the Safety and Efficacy of Herbal Medicines.

In skeletal muscles, antioxidants are enzymatic (e.g., Glutathione peroxidase (GPx) and catalase) and nonenzymatic (for example, GSH, uric acid, bilirubin, vitamin E, vitamin C, etc.) function as an integrated antioxidant complex that acts to capture ROS. ^27^ ^,^ ^28^ These intracellular antioxidants are usually present in cells, cytoplasm, and organelles (for example, mitochondria) whose role is to protect muscle fibres from damage caused by ROS. ^27^ ^,^ ^29^ ^–^ ^31^ Endogenous free radicals are formed as a normal response to the chain reaction of respiration in the body. The free radicals in the body are balanced by an endogenous defense system mechanism, ^32^ in which the body produces antioxidants that have an anti-free radical effect. One of the endogenous antioxidants is SOD, which is the body's first line of defense against ROS activation. ^8^ When the level of ROS rises beyond the endogenous defense capacity, oxidative instability, known as oxidative stress, occurs. ^9^ ^,^ ^29^ Oxidative stress conditions due to free radicals will cause lipid peroxidation of cell membranes and damage cell membrane organization. One of the biomarkers of oxidative stress is a high level of MDA ^33^ and decreased SOD activity due to excessive lipid peroxidation processes in cells. ^9^ One way to control excessive oxidative stress is by consuming antioxidants from food (exogenous antioxidants). ^34^ One source of exogenous antioxidants is RDF that can be found in Indonesia.

In this study, the endogenous antioxidants in the body were superoxide dismutase (SOD), and they are unable to neutralize free radicals. This condition results in an imbalance of free radicals and antioxidants, leading to oxidative damage, as reported in previous studies. ^35^ Unstabilized oxidative stress produces free radicals, which can damage muscle tissue and lungs and cause impaired cell function, which is involved in muscle fatigue. RDF treatment can increase SOD significantly (p<0.05) and function as a good source of several natural antioxidants, such as betalain, polyphenols, and ascorbic acid, as evidenced in previous studies. ^36^ ^,^ ^37^ During strenuous exercise, the increase in ROS formation during contractile activity is directly related to increased oxygen consumption. This condition results in a 50 or 100 fold increase in mitochondrial activity in the formation of superoxide in skeletal muscle during aerobic contraction. ^38^ ^,^ ^39^ An increase in oxidative stress, as observed, leads to an increase in lipid peroxidation accompanied by a decline in SOD level activity, as the antioxidants are given depending on the dose affect the increase in SOD levels. ^28^ This improvement in oxidative status suggests that the natural antioxidants in the extract with high doses were responsible for delaying fatigue in this study, as reported in previous studies. ^40^ ^,^ ^41^ In this study, it was found that the higher the dose given, the greater the SOD, as shown in group P3 that was on treatment so that this SOD level could neutralize free radicals. The SOD enzyme is the first defense system against free radicals. Thus, moderate-intensity regular exercise has been shown to increase antioxidant defenses by increasing the activity of endogenous antioxidant enzymes, such as SOD, glutathione peroxidase, and catalase. ^42^ ^,^ ^43^ These enzymes can suppress or inhibit the formation of free radicals by breaking the chain reaction so that the product is more stable. This process is known as the antioxidant chain-breaking reaction.

RDF is rich in antioxidants, such as phenol and flavonoid compounds. Phenolic compounds that function as antioxidants neutralize free radicals and peroxide radicals to inhibit lipid oxidation effectively. Flavonoids are exogenous antioxidants that are beneficial in preventing cell damage due to oxidative stress. Its role is to donate hydrogen ions to neutralize the toxic effects from free radicals due to exercise. RDF consumption can also increase the VO2max value. ^44^

The relationship between the provision of antioxidants after treatment with RDF extract is that the administration of exogenous antioxidants helps suppress the spread of free radicals in the body because antioxidants can come from within the body (endogenous) or come from outside the body (exogenous), simultaneously suppressing free radicals due to exercise.

Anthocyanin is one type of flavonoid widely found in dragon fruit, ^45^ which is able to improve mitochondrial function by influencing free radicals. Anthocyanins can suppress the occurrence of lipid peroxidation as an inflammatory response due to free radicals, thereby suppressing the production of MDA. ^46^

An increase in the free radicals in the body causes an imbalance between oxidants and antioxidants. This condition leads to oxidative stress. The earliest known and widely studied cell or tissue mechanism is lipid peroxidation. RDF extract contains anthocyanin pigments which function as antioxidants. ^18^ ^,^ ^47^ ^,^ ^48^ Anthocyanins can play a role in inhibiting free radicals that occur due to strenuous exercise. This study examined the provision of RDF extract comprising anthocyanins, one of the types contained in flavonoids, which provides a response to inflammation in the muscles and lung tissue. The presence of anthocyanins repairs damaged tissue so that physiological mitochondrial function returns, as anthocyanins can suppress the occurrence of lipid peroxidation and suppress MDA production so that MDA levels decrease. ^49^ ^,^ ^50^ Anthocyanins can quickly bind metal ions to form a stable anthocyanin-metal complex. This means that anthocyanins bind to the transitioned ion metal to prevent highly toxic and reactive hydroxyl reactions. In the end, anthocyanins can suppress lipid peroxidation and suppress MDA production to reduce MDA levels.

## Conclusions

Strenuous exercise causes an increase in ROS, resulting in increased free radical levels, leading to oxidative stress to occur. Ingesting RDF extracts suppresses the increase. The group that was given RDF doses of 150 mg, and 300 mg performed better than the group with a dose of 75 mg in responding to oxidative stress with strenuous exercise. RDF extract dose resulted in decreased oxidative stress, repaired muscle and lung tissue.

## Data availability

### Underlying data

Figshare: Datasets, https://doi.org/10.6084/m9.figshare.15074544.v5. ^51^ This project contains the following underlying data:

- MDA RAT.xls (MDA levels for all groups)
- SOD RAT 23 Maret 21.xls (SOD levels for all groups)
- Table HEnew.docx (scoring for microscopy results)

## Reporting guidelines

Figshare: ARRIVE checklist for ‘The effect of ingestion of Red dragon fruit extract on levels of malondialdehyde and superoxide dismutase after strenuous exercise in rats ( *Rattus norvegicus*)’, https://doi.org/10.6084/m9.figshare. 15074544.v5. ^51^

Data are available under the terms of the Creative Commons Attribution 4.0 International license (CC-BY 4.0).
