# Supplementary material for: The effect of ingestion of red dragon fruit extract on levels of malondialdehyde and superoxide dismutase after strenuous exercise in rats ( Rattus norvegicus)
Source: F1000Res. 2022 Aug 4;10:1061. Originally published 2021 Oct 18. [Version 3] doi: 10.12688/f1000research.54254.3 (PMC9345267; doi:10.12688/f1000research.54254.3)
Supplement: Peer review history

# Peer review: Reviewer response for version 3

**DOI:** 10.5256/f1000research.137030.r146521

**Published:** 2022-08-05

**Authors:** Ermita I. Ibrahim Ilyas

**Affiliations:** 1 Faculty of Medicine, Department of Medical Physiology, Universitas Indonesia, Jakarta, Indonesia

The minor correction has been done.

Is the work clearly and accurately presented and does it cite the current literature?

Partly

If applicable, is the statistical analysis and its interpretation appropriate?

Yes

Are all the source data underlying the results available to ensure full reproducibility?

Partly

Is the study design appropriate and is the work technically sound?

Partly

Are the conclusions drawn adequately supported by the results?

No

Are sufficient details of methods and analysis provided to allow replication by others?

Partly

Reviewer Expertise:

NA

I confirm that I have read this submission and believe that I have an appropriate level of expertise to confirm that it is of an acceptable scientific standard.

# Peer review: Reviewer response for version 2

**DOI:** 10.5256/f1000research.135241.r143820

**Published:** 2022-08-01

**Authors:** Ermita I. Ibrahim Ilyas

**Affiliations:** 1 Faculty of Medicine, Department of Medical Physiology, Universitas Indonesia, Jakarta, Indonesia

The authors have satisfactorily addressed my concerns, so I approve this article. However, there are still some minor corrections needed, which I have highlighted in the attached manuscript.

Is the work clearly and accurately presented and does it cite the current literature?

Partly

If applicable, is the statistical analysis and its interpretation appropriate?

Yes

Are all the source data underlying the results available to ensure full reproducibility?

Partly

Is the study design appropriate and is the work technically sound?

Partly

Are the conclusions drawn adequately supported by the results?

No

Are sufficient details of methods and analysis provided to allow replication by others?

Partly

Reviewer Expertise:

NA

I confirm that I have read this submission and believe that I have an appropriate level of expertise to confirm that it is of an acceptable scientific standard.

# Peer review: Reviewer response for version 2

**DOI:** 10.5256/f1000research.135241.r143819

**Published:** 2022-07-11

**Authors:** Farzaneh Taghian

**Affiliations:** 1 Department of Exercise Physiology, Faculty of Sport Sciences, University of Isfahan, Isfahan, Iran

This paper sounds good. 

 Best wishes

Is the work clearly and accurately presented and does it cite the current literature?

No

If applicable, is the statistical analysis and its interpretation appropriate?

Yes

Are all the source data underlying the results available to ensure full reproducibility?

Yes

Is the study design appropriate and is the work technically sound?

No

Are the conclusions drawn adequately supported by the results?

No

Are sufficient details of methods and analysis provided to allow replication by others?

No

Reviewer Expertise:

Sport and nutrition

I confirm that I have read this submission and believe that I have an appropriate level of expertise to confirm that it is of an acceptable scientific standard.

# Peer review: Reviewer response for version 1

**DOI:** 10.5256/f1000research.57721.r137352

**Published:** 2022-06-07

**Authors:** Ermita I. Ibrahim Ilyas

**Affiliations:** 1 Faculty of Medicine, Department of Medical Physiology, Universitas Indonesia, Jakarta, Indonesia

This study was quite informative. There are some suggestions to improve this article:

1. The title should be in accordance with the result of the study.
2. Abstract should be rewritten since there were information missed in the methods, results and conclusion. Please refer to the main body of the manuscript.
3. The introduction also needs to be rewritten to make it clear, focused and concise to the aim of the study. Authors have to provide a comprehensive description

Methods:

- Study design has to explain the grouping of the rats with proper terms.
- Experimental procedures in the main manuscript were different than in the abstract.
- Outcome: authors mentioned about repair mitochondrial cell function has fatigue delaying effect. However, authors did not analyse or measure the mitochondria function and fatigue duration and time.

Results:

- Write complete legends of the figures including clear explanation of the sign.
- Explain how to determine the improvement of lung and muscle cells (histopathological change in muscle and lung tissue) in the rats supplemented by RDF.
- How to determine the fatigue? Authors mentions the fatigue delay with no data showing this.

Discussion:

- Rewrite to make it systematic, focused and clear to provide a comprehensive report.

Conclusion:

- My answer to the question, "Are the conclusions drawn adequately supported by the results?" is 'No' because the conclusion of the manuscript was not written adequately from the result of the study. My suggestion is that they have to write the conclusion based on all the parameters they analyzed. 

**Conclusion in the abstract:**

*"This study confirmed that strenuous exercise that causes an increase in ROS intensifies free radicals with RDF extract ingestion and declines stress oxidative, repairing cell function and delaying fatigue."*

**My suggestion for Conclusion in the abstract:**

 "This study confirmed that strenuous exercise causes an increase in ROS intensifies free radicals shown by MDA increase and SOD decrease, and with RDF extract ingestion MDA decrease and SOD increase showed RDF declines stress oxidative, and 300 mg kg ^-1^.bw of RDF extract showed better result compared to other doses. The decrease of oxidative stress, showed improvement of muscle and lung tissue injuries." 

**Conclusion in the main body of the manuscript:**

*"Strenuous exercise causes an increase in ROS, resulting in increased free radical levels, leading to oxidative stress to occur. Ingesting RDF extracts suppresses the increase. The group was given RDF doses of 150 mg, and 300 mg performed better than 75 mg in responding to oxidative stress with strenuous exercise. This condition results in decreased oxidative stress, repaired muscle and lung tissue, so that cell function returned physiologically, which delayed fatigue."*

**My suggestion for Conclusion in the main body of manuscript:**

 "Strenuous exercise causes an increase in ROS, resulting in increased free radical levels, leading to oxidative stress to occur shown by MDA increase and SOD decrease. Ingesting RDF extracts suppresses the increase of oxidative stress. The group that was given RDF doses of 150 mg and 300 mg performed better than 75 mg in responding to oxidative stress. The decrease of oxidative stress, showed improvement of muscle and lung tissue injuries." 

**Note: **

- The manuscript's language and grammar should undergo a thorough proofreading.
- Authors should rewrite the manuscript based on reviewer’s suggestions.

Is the work clearly and accurately presented and does it cite the current literature?

Partly

If applicable, is the statistical analysis and its interpretation appropriate?

Yes

Are all the source data underlying the results available to ensure full reproducibility?

Partly

Is the study design appropriate and is the work technically sound?

Partly

Are the conclusions drawn adequately supported by the results?

No

Are sufficient details of methods and analysis provided to allow replication by others?

Partly

Reviewer Expertise:

Exercise Physiology

I confirm that I have read this submission and believe that I have an appropriate level of expertise to confirm that it is of an acceptable scientific standard, however I have significant reservations, as outlined above.

# Response

**Published:** 2022-06-21

**Authors:** Prof Gusbakti Rusip

**Affiliations:** Universitas Prima Indonesia, Indonesia

It has been a great sense of delight to receive your very friendly and professional comments. We want to thank the reviewer for the time and willingness to assess the quality of our manuscript. In the new version, our manuscript suggested changes in all sections have been added according to the reviewer's recommendation.

 Kindest regards,

 Prof Dr Gusbakti Rusip, MD, Sp.KKLP, AIFM, Department Physiology, University Prima Indonesia

1. Introduction
  One source of natural antioxidants is red dragon fruit peel ( *Hylocereus polyrhizus*). Red dragon fruit peel (1 mg/kg) is able to inhibit 83.48% of free radicals. Red dragon fruit peels has antioxidant activity (IC50 = 43,836 microgram/mL). The antioxidant of red dragon fruit peel is higher than its flesh. Red dragon fruit peel contains a natural antioxidant compound of phenolic or polyphenolic. It has antioxidant activity of flavones, flavonols, isoflavones, catechins and kalkon. Red dragon fruit peel also contains betacyanin. Betacyanin is part of the pigment betalains and has antioxidant properties (neutralizing free radicals). Betacyanin of red dragon fruit peel, including phenolic compounds. Phenolic derivatives or polyphenols (as antioxidants) stabilize free radicals by supplementing lack of electrons possessing free radicals and inhibiting the occurrence of chain reactions from free radical formation.
2. Methods and materials
  In this study, we used 25 three-month-old male rats with an average weight of 200 g. The rats were obtained from the Animal House Unit of the Biology Laboratory, Universitas Sumatera Utara, Indonesia. All rats were maintained in groups in experimental animal cages in the laboratory. The cage (30 cm, 20 cm, 10 cm) was made of plastic and covered with fine wire mesh. The cage base was covered with rice husks with a thickness of 0.5–1 cm, which was replaced daily during the study. The room light was controlled to deliver a 12h light/12h dark cycle, the temperature was set to 25–27 °C, and the room's humidity was adjusted to a normal range of 35–50%. The rats were fed standard rat pellets and given tap water *ad libitum*.
3. Design study
  We used an in vitro experimental method with an actual experimental design and a randomised post-test for the control group. Simple random sampling was used to categorise the laboratory rats into five groups as follows: group K1 with no activity and no RFP; group K2 subjected to strenuous exercise without RDF (Red Dragon Fruit); and groups P1, P2, and P3 subjected to strenuous exercise and treated with 75, 150, and 300 mg kg ^−1^ body weight of RDF extract, respectively. In the fruit market, it is easy to find RFP fruit acquired from farmers in Indonesia, peeled, washed, cut into small pieces, and then dried in a drying cabinet. Next, the fruit was blended using a blender, and the extract was obtained by the maceration method with 96% ethanol, which was distilled by ten times the weight of RDF. The RDF powder was stored in a container with 96% ethanol (ratio of 1:7, fruit powder: ethanol) and then soaked for 3 d. The RDF was macerated using a rotary evaporator at 45 °C until the extract thickened. The macerated RDF was extracted using 96% ethanol. The remaining extract was evaporated in a water bath until a thick extract was obtained. Next, 100 mg RDF extract was weighed and crushed using a pestle and mortar. Subsequently, carboxymethylcellulose Na solution (0.5% w/v) was slowly added until a homogeneous extract was obtained, and the resulting volume was 10 mL. This final RFP extract was administered to the rats at appropriate dosages; specifically, rats weighing 200 g were fed 1.5, 3.0, or 6.0 mL of the RFP extract suspension, corresponding to doses of 75, 150, or 300 mg kg ^−1 ^body weight, respectively.
4. Analysis of blood samples
  All rats performed strenuous exercise until they reached their maximum effort (i.e., swimming until they almost drowned). At this time, blood samples were sequentially taken to analyse malondialdehyde (MDA) and SOD using the enzyme-linked immune sorbent assay (ELISA) method with spectrophotometry at a wavelength of 450 nm. The mouse malondialdehyde ELISA kit (Brand Bioassay TL, catalog: EO625Mo) was used to analyse the MDA levels. The SOD kit (Brand Bioassay TL, catalog: EO168Ra) Rat super Oxidase Dismutase ELISA kit was determined using the equation obtained from the standard curve.
5. Histopathological study
  Muscle and lung tissue samples were collected by performing a biopsy to determine the degree of muscle damage based on haematoxylin and eosin (H&E) staining. The soleus muscle tissues of the rats were collected and fixed with 10% formalin for 24 h. The muscle and lung tissues were embedded in paraffin, sectioned to a 4 μm thickness, and stained via H&E staining. The stained sections were then examined under a light microscope (400' magnification) with 10 fields of view to determine the degree of damage concerning inflammatory cells and necrosis. The examination was conducted by a pathologist who was blinded to the applied treatment.

# Response

**Published:** 2022-06-21

**Authors:** Prof Gusbakti Rusip

**Affiliations:** Universitas Prima Indonesia, Indonesia

We would like to thank the reviewer for the time and willingness to assess the quality of our manuscript. In the new version, the suggested changes in all sections have been added according to the reviewers recommendation. It has been a great sense of delight to receive your very nice and professional comments.

 Kindest regards,

 Prof Dr Gusbakti Rusip, MD, Sp.KKLP,AIFM, Department Physiology, University Prima Indonesia

1. The title should be in accordance with the result of the study.
*We agree that we have adjusted the results of the study.*
2. Abstract should be rewritten since there were information missed in the methods, results and conclusion. Please refer to the main body of the manuscript.
*The manuscript has been revised and has been completed.*
3. The introduction also needs to be rewritten to make it clear, focused and concise to the aim of the study. Authors have to provide a comprehensive description.
*The manuscript has been revised and has been completed.*

Methods:

1. Study design has to explain the grouping of the rats with proper terms. Experimental procedures in the main manuscript were different than in the abstract.
*It has been revised according to the research objectives and has been perfected.*
2. Outcome: authors mentioned about repair mitochondrial cell function has fatigue delaying effect. However, authors did not analyse or measure the mitochondria function and fatigue duration and time.
*Mitochondrial function analysis has not been analyzed, the research is in progress.*

Results:

1. Write complete legends of the figures including clear explanation of the sign.
*We have fixed it in the revised article.*
2. Explain how to determine the improvement of lung and muscle cells (histopathological change in muscle and lung tissue) in the rats supplemented by RDF.
*Monitoring the repair of muscle and lung cells seen from the number of inflammatory cells and necrosis then examined under a light microscope (400x magnification) with ten fields of view to ascertain the degree of damage with respect to inflammatory cells and necrosis seen in Fig share table HE.*
3. How to determine the fatigue? Authors mentions the fatigue delay with no data showing this.
*The results of this study are that we write down the time of fatigue before and after giving Red Dragon Fruit after we have entered the data on the results of the comparison.*

Discussion:

- Rewrite to make it systematic, focused and clear to provide a comprehensive report.
*We have systematically improved and completed several discussions for the perfection of the article.*

Conclusion:

- My answer to the question, "Are the conclusions drawn adequately supported by the results?" is 'No' because the conclusion of the manuscript was not written adequately from the result of the study. My suggestion is that they have to write the conclusion based on all the parameters they analyzed.
*We have adjusted the conclusion to the topic, we found the results of our research.*

# Peer review: Reviewer response for version 1

**DOI:** 10.5256/f1000research.57721.r127975

**Published:** 2022-03-29

**Authors:** Farzaneh Taghian

**Affiliations:** 1 Department of Exercise Physiology, Faculty of Sport Sciences, University of Isfahan, Isfahan, Iran

The author evaluated the effect of ingestion of red dragon fruit extract on levels of malondialdehyde and superoxide dismutase after strenuous exercise in rats. I think this study was informative and practical.

 However, there are some comments to improve this study:

- In the Introduction section explain red dragon fruit in detail.
- The grouping is not clear. Describe it. What is N, A, or RDF? Completely explain the grouping.
- Study design is not suitable. The author has to explain the standard condition of the rats and grouping.
- Write the commercial name of the kits, catalog number.
- The grammar and writing is poor
- Write the protocol of Hematoxylin Eosin (H&E) staining
- The quality of the image is poor.

Is the work clearly and accurately presented and does it cite the current literature?

No

If applicable, is the statistical analysis and its interpretation appropriate?

Yes

Are all the source data underlying the results available to ensure full reproducibility?

Yes

Is the study design appropriate and is the work technically sound?

No

Are the conclusions drawn adequately supported by the results?

No

Are sufficient details of methods and analysis provided to allow replication by others?

No

Reviewer Expertise:

Sport and nutrition

I confirm that I have read this submission and believe that I have an appropriate level of expertise to confirm that it is of an acceptable scientific standard, however I have significant reservations, as outlined above.

# Response

**Published:** 2022-04-05

**Authors:** Prof Gusbakti Rusip

**Affiliations:** Universitas Prima Indonesia, Indonesia

We would like to thank the reviewer for the time and willingness to assess the quality of our manuscript. In the new version of our manuscript, suggested changes in all sections have been added according to the reviewer’s recommendation. It has been a great delight to receive your very nice and professional comments.

 Kindest regards,

 Prof Dr Gusbakti Rusip, MD, Sp.KKLP,AIFM, Department Physiology, University Prima Indonesia

1. Introduction: One source of natural antioxidants is red dragon fruit peel ( *Hylocereus polyrhizus*). Red dragon fruit peel (1 mg/kg) can inhibit 83.48% of free radicals. Red dragon fruit peels has antioxidant activity (IC50 = 43,836 microgram/mL). The antioxidants of red dragon fruit peel are higher than in its flesh. Red dragon fruit peel contains natural antioxidant compounds in phenolic or polyphenolic. It has antioxidant activity of flavones, flavonols, isoflavones, catechins and kalkon.  Red dragon fruit peel also contains betacyanin. Betacyanin is part of the pigment betalains and has antioxidant properties (neutralizing free radicals). Betacyanin of red dragon fruit peel including phenolic compounds. Phenolic derivatives or polyphenols (as antioxidants) stabilize free radicals by supplementing lack of electrons possessing free radicals and inhibiting the occurrence of chain reactions from free radical formation.
2. Methods and Materials: In this study, we used 25 three-month-old male rats with an average weight of 200 g. The rats were obtained from the Animal House Unit of the Biology Laboratory, Universitas Sumatera Utara, Indonesia. All rats were maintained in groups in experimental animal cages in the laboratory. The cage (30 cm,20 cm, 10 cm) was made of plastic and covered with fine wire mesh. The cage base was covered with rice husks with a thickness of 0.5–1 cm, which was replaced every day during the study. The room light was controlled to deliver a 12 h light/12 h dark cycle, the temperature was set to 25–27 °C, and the room's humidity was adjusted to a normal range of 35–50%. The rats were fed standard rat pellets and given tap water *ad libitum*.
3. Design study: We used an in vitro experimental method with a true experimental design and a randomised post-test for the control group. Simple random sampling was used to categorise the laboratory rats into five groups as follows: group K1 with no activity and no RDF; group K2 subjected to strenuous exercise without RDF (Red Dragon Fruit); and groups P1, P2, and P3 subjected to strenuous exercise and treated with 75, 150, and 300 mg kg ^−1^ body weight of RDF extract, respectively. It is easy to find RDF in the fruit market, acquired from farmers in Indonesia, peeled, washed, cut into small pieces, and then dried in a drying cabinet. Next, the fruit was blended using a blender, and the extract was obtained by the maceration method with 96% ethanol, which was distilled by 10 times the weight of RDF. The RDF powder was stored in a container with 96% ethanol (ratio of 1:7, fruit powder: ethanol) and then soaked for 3 d. The RDF was macerated using a rotary evaporator at 45 °C until the extract thickened. The macerated RDF was extracted using 96% ethanol. The remaining extract was then evaporated in a water bath until a thick extract was obtained. Next, 100 mg RDF extract was weighed and crushed using a pestle and mortar. Subsequently, carboxymethylcellulose Na solution (0.5% w/v) was slowly added until a homogeneous extract was obtained, and the resulting volume was 10 mL. This final RDF extract was administered to the rats at appropriate dosages; specifically, rats weighing 200 g were fed 1.5, 3.0, or 6.0 mL of the RDF extract suspension, which corresponded to doses of 75, 150, or 300 mg kg ^−1 ^body weight, respectively.
4. Analysis of blood samples: rats performed strenuous exercise until they reached their maximum effort (i.e., swimming until they almost drowned). At this time, blood samples were sequentially taken to analyse malondialdehyde (MDA) and SOD using the enzyme-linked immune sorbent assay (ELISA) method with spectrophotometry at a wavelength of 450 nm. The mouse malondialdehyde ELISA kit (Brand Bioassay TL, catalog: EO625Mo) was used to analyse the MDA levels. The SOD kit ( Brand Bioassay TL, catalog: EO168Ra) Rat super Oxidase Dimutase ELISA kit was determined using the equation obtained from the standard curve.
5. Histopathological study muscle and lung tissue samples were collected by performing a biopsy to determine the degree of muscle damage based on haematoxylin and eosin (H&E) staining. The soleus muscle tissues of the rats were collected and fixed with 10% formalin for 24 h. The muscle and lung tissues were embedded in paraffin, sectioned to a 4 μm thickness, and stained via H&E staining. The stained sections were then examined under a light microscope (400X magnification) with 10 fields of view to determine the degree of damage concerning inflammatory cells and necrosis. The examination was conducted by a pathologist blinded to the applied treatment.
